# Archazolid and apicularen: Novel specific V-ATPase inhibitors

**DOI:** 10.1186/1471-2091-6-13

**Published:** 2005-08-04

**Authors:** Markus Huss, Florenz Sasse, Brigitte Kunze, Rolf Jansen, Heinrich Steinmetz, Gudrun Ingenhorst, Axel Zeeck, Helmut Wieczorek

**Affiliations:** 1 Universität Osnabrück, Fachbereich Biologie/Chemie, Abteilung Tierphysiologie, 49069 Osnabrück, Germany; 2 Gesellschaft für Biotechnologische Forschung, Bereich Naturstoffe, 38124 Braunschweig, Germany; 3 Universität Göttingen, Fakultät für Chemie, Institut für Organische und Biomolekulare Chemie, 37077 Göttingen, Germany

## Abstract

**Background:**

V-ATPases constitute a ubiquitous family of heteromultimeric, proton translocating proteins. According to their localization in a multitude of eukaryotic membranes, they energize many different transport processes. Since their malfunction is correlated with various diseases in humans, the elucidation of the properties of this enzyme for the development of selective inhibitors and drugs is one of the challenges in V-ATPase research.

**Results:**

Archazolid A and B, two recently discovered cytotoxic macrolactones produced by the myxobacterium *Archangium gephyra*, and apicularen A and B, two novel benzolactone enamides produced by different species of the myxobacterium *Chondromyces*, exerted a similar inhibitory efficacy on a wide range of mammalian cell lines as the well established plecomacrolidic type V-ATPase inhibitors concanamycin and bafilomycin. Like the plecomacrolides both new macrolides also prevented the lysosomal acidification in cells and inhibited the V-ATPase purified from the midgut of the tobacco hornworm, *Manduca sexta*, with IC_50 _values of 20–60 nM. However, they did not influence the activity of mitochondrial F-ATPase or that of the Na^+^/K^+^-ATPase. To define the binding sites of these new inhibitors we used a semi-synthetic radioactively labelled derivative of concanamycin which exclusively binds to the membrane V_o _subunit c. Whereas archazolid A prevented, like the plecomacrolides concanamycin A, bafilomycin A_1 _and B_1_, labelling of subunit c by the radioactive I-concanolide A, the benzolactone enamide apicularen A did not compete with the plecomacrolide derivative.

**Conclusion:**

The myxobacterial antibiotics archazolid and apicularen are highly efficient and specific novel inhibitors of V-ATPases. While archazolid at least partly shares a common binding site with the plecomacrolides bafilomycin and concanamycin, apicularen adheres to an independent binding site.

## Background

Vacuolar-type ATPases (V-ATPases) are ubiquitous proton pumps in the endomembrane system of all eukaryotic cells and in plasma membranes of many animal cells where they energize transport processes across the membrane or regulate the pH of corresponding compartments [1]. They are heteromultimeric enzymes consisting of a membrane bound, proton translocating V_o _complex and a catalytic V_1 _complex which is oriented towards the cytosol. In recent years it became more and more evident that malfunction of the V-ATPase is correlated with a multitude of diseases such as osteopetrosis, male infertility or renal acidosis [2-4]. Therefore the V-ATPase turned out to be a subject for biomedical research and even was considered as a potential target for cancer drug therapy [5]. In order to understand the development of these diseases and to design efficient drugs for their therapy it is necessary to gain a most comprehensive knowledge of the mode of action of the enzyme as well as of known V-ATPase inhibitors on the one hand, and, on the other hand, to search for novel potent and specific inhibitors with different inhibition characteristics.

The best examined and established specific V-ATPase inhibitors are the plecomacrolides bafilomycin [6] and concanamycin [7], which both take effect in nanomolar concentrations by binding to the V_o _subunit c [8-10]. Recently various new inhibitors of V-ATPases such as the benzolactone enamides [11] or chondropsines [12] have been described (reviewed in [13]) but so far in no case the binding site has been determined. Only for the benzolactone enamide salicylihalamide it was shown that its binding site is different to that of plecomacrolides [10] and may reside somewhere between the V_o _and the V_1 _complex [14]. In the present report we introduce two types of antibiotics produced by myxobacteria, apicularens, new benzolactone enamides [15,16] and archazolids, a novel class of macrolactones [17] which both represent highly potent and specific V-ATPase inhibitors, however, with different modes of action and different binding sites.

## Results and Discussion

### Archazolid and apicularen influence the viability of mammalian cell-lines

**Figure 1:**
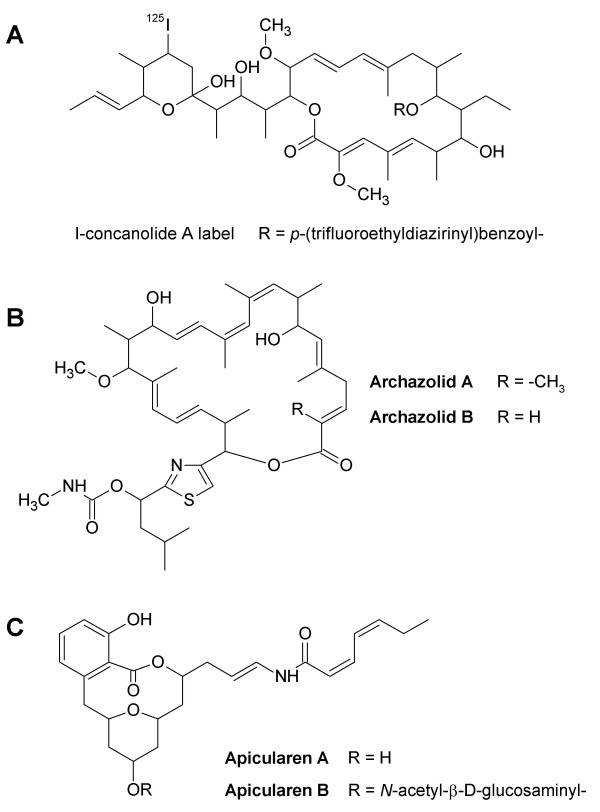
**Structure of the antibiotics**. A, I-concanolide A (9-*O*-[p-(trifluoroethyldiazirinyl)-benzoyl]-21,23-dideoxy-23-[^125^I]iodo-concanolide A). B, archazolid A and B. C, apicularen A and B.

**Figure 2:**
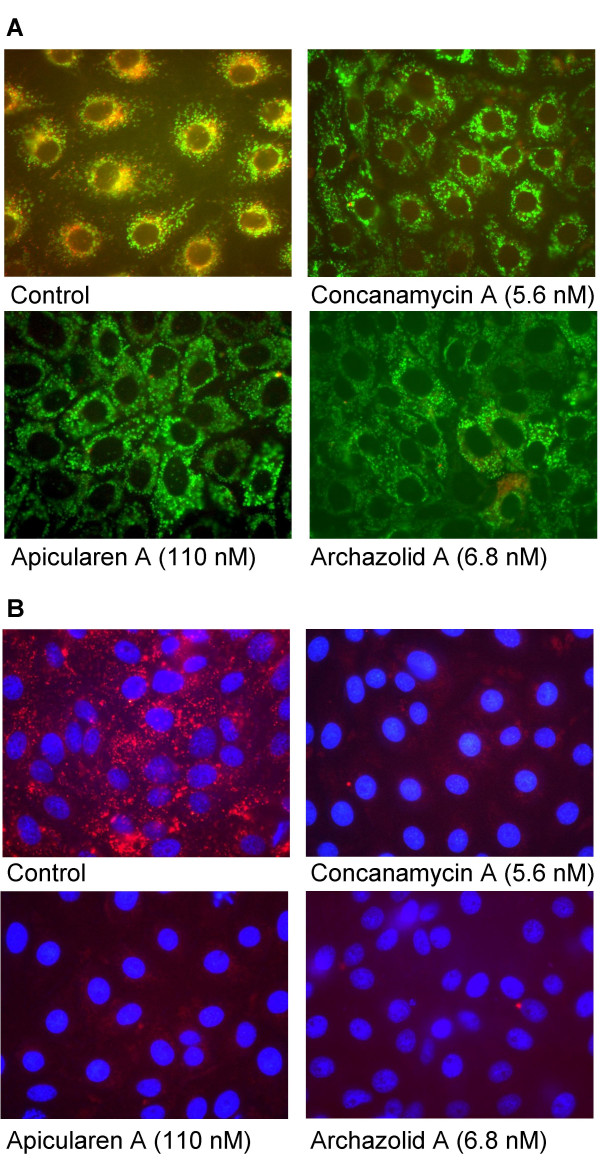
**Inhibition of lysosomal acidification by the novel inhibitors**. Potoroo kidney cells (PtK_2_) were treated with V-ATPase inhibitors for 4 hours and stained for lysosomes (red) with the acidotropic reagent LysoTracker, for mitochondria with MitoTracker (A, green) or for nuclei with Hoechst 33258 (B, blue). The control cells show many red vesicles indicating acidic lysosomes whereas in cells treated with the inhibitors only few red spots can be observed.

The novel antibiotics archazolids and apicularens (Fig. 1) were checked for their impact on the cell growth of a variety of mammalian cell lines from different tissues (Tab. 1). For nearly all of them IC_50 _values were in the nanomolar range, comparable with the IC_50 _values for concanamycin A and bafilomycin A_1_. Apicularen B was the only exception, with an average IC_50 _value two orders of magnitude higher. Growth inhibition of the multidrug-resistant cell line KB-V1 was also measured in the presence of verapamil. As this compound inactivates the Pgp efflux pump, a comparison of the IC_50 _values obtained in the presence and in the absence of verapamil revealed to which extent the compounds were pumped out of the cells by the MDR1 Pgp. The data in Tab 1. show, that unlike the archazolids, the apicularens are poor substrates of Pgp. To visualize the impact of the antibiotics, PtK_2 _(potoroo kidney) cells were incubated with the inhibitors and stained for intact acidic lysosomes (Fig. 2). Evidently, in the presence of apicularen A and archazolid A as well as in the presence of concanamycin A and of bafilomycin A_1 _(not shown) the red staining, indicating acidic lysosomes, disappeared compared to cells which had not been treated by drugs. The same observation was made with KB-3-1 cells (data not shown). These results provided the first indication that the novel antibiotics, like concanamycin or bafilomycin, are interfering with the V-ATPase which is the obligatory acidifier of lysosomes. Unlike the cell lines listed in Tab. 1, the growth of PtK_2 _cells was not completely stopped. The same holds true for A-498 cells (human kidney carcinoma). Both cell lines grow epithelial-like. There was obviously a differential reaction of cell lines to V-ATPase inhibitors. For A-431 cells (human epidermoid carcinoma) a dependence on the expression of the EGF receptor was shown [18].

**Table 1:** Growth inhibition of different mammalian cell lines by antibiotics

Cell line	Origin	ArcA	ArcB	ApiA	ApiB	BafA_1_	ConA
		IC_50 _(nM)					
L-929	murine connective tissue	0.81^a^	1.1^a^	4.5	620	3.2^a^	0.23^a^
3Y1	rat, embryogenic fibroblast cell line	0.95	1.1	3.2	310	5.6	1.4
KB-V1^b^	human cervix carcinoma	48.0	35.0	23.0	1600	7.2	28.0
KB-V1^b^	(in presence of 11 μM verapamil)	2.3	1.5	7.9	540	4.0	2.7
A-594	human lung carcinoma	0.54	0.69	0.23^c^	310^c^	0.4	0.17
M1	mouse, embryogenic fibroblast cell line	0.27	0.35	1.4	190	2.6	0.56

^a ^[17]; ^b ^mdr cell line; ^c ^[15]

DSMZ: L-929, KB-V1, A-594

3Y1: [33]

M1: [30]

### The V-ATPase is highly sensitive to archazolid and apicularen

**Figure 3:**
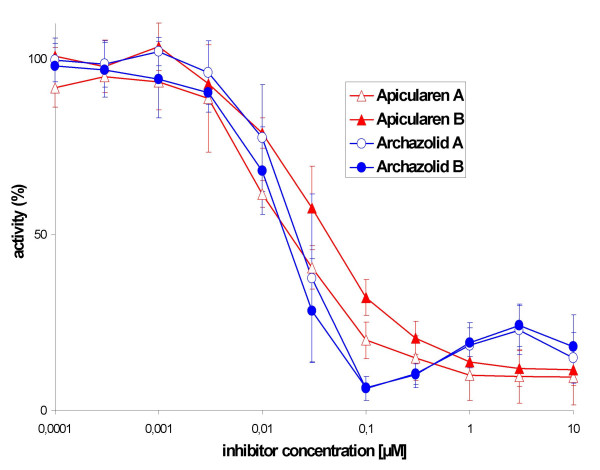
**Inhibition of the V_1_/V_o _holoenzyme activity by the antibiotics. **Values represent the means ± S.D. of three independent enzyme preparations. Archazolid A (open circles), archazolid B (solid circles), apicularen A (open triangles) and apicularen B (solid triangles). The specific enzyme activity of the controls without inhibitors was 1.5 ± 0.2 μmol*mg^-1 ^*min^-1^.

To verify our assumption, we tested the inhibitory efficacy of archazolid A and B as well as of apicularen A and B on the purified V-ATPase holoenzyme from the midgut of the tobacco hornworm. As shown in Fig. 3, both archazolids inhibited the purified enzyme half-maximally at a concentration of ca. 20 nM, equivalent to an IC_50 _value of ca. 0.8 nmol per mg of protein. Apicularen A and B inhibited the purified enzyme half-maximally at concentrations of ca. 20 nM and 60 nM, respectively, equivalent to IC_50 _values of ca. 0.8 nmol and 2.4 nmol per mg of protein. These inhibitory values were in the same concentration range as those measured for the plecomacrolides concanamycin A, bafilomycin A_1 _and B_1_, and of the benzolactone enamide salicylihalamide which all exhibited a half-maximal inhibition at ca. 10 nM and an IC_50 _of ca. 0.5 nmol per mg of protein [10]. Thus the novel compounds are highly efficient V-ATPase inhibitors.

These findings confirmed our assumption that growth inhibition and the morphological changes induced by archazolid and by apicularen were due to their inhibitory effect on the V-ATPase. Apicularen B was slightly lower active compared to the other drugs, but the difference is much less than expected from the cell culture assays. This difference may result from a lower membrane permeability of apicularen B due to its additional *N*-acetyl-*β*-D-glucosamine residue.

While the macrolactones (archazolid A and B) and the benzolactone enamides (apicularen A and B, salicylihalamide) were shown to be potent growth inhibitors of cultured mammalian cells, they all failed to inhibit bacterial growth, while only archazolid showed weak activity against fungi; in addition, the benzolactone enamide salicylihalamide effectively inhibited mammalian V-ATPases but not at all V-ATPases from fungi such as *Saccharomyces cerevisiae *or *Neurospora crassa *[11,15,17]. Since the tobacco hornworm V-ATPase is very sensitive to all these antibiotics and available in milligram amounts, it appears to be an appropriate model for further investigations on the mechanism of inhibition by archazolid and the benzolactone enamides.

### Archazolid and apicularen do not appear to inhibit F-and P-ATPases

**Figure 4:**
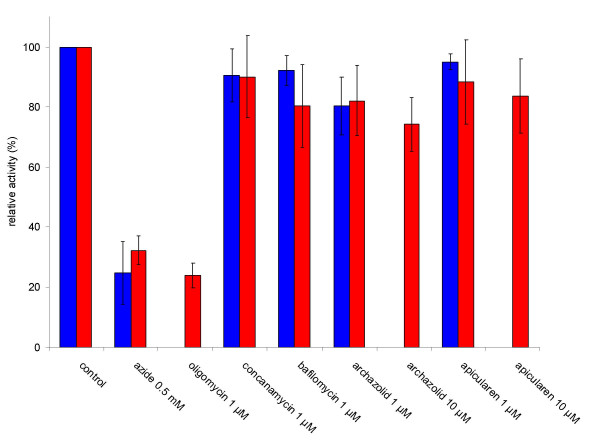
**Inhibition of F-ATPase activity in crude membranes from mouse heart and submitochondrial particles from beef heart**. Values represent the means ± S. D. of three (mouse heart) or four (beef heart) independent experiments., respectively. The specific ATPase activity without inhibitor was 0.16 ± 0.05 μmol*mg^-1 ^*min^-1 ^in mouse heart crude membranes (blue columns) and 6.1 ± 0.35 μmol*mg^-1 ^*min^-1 ^in submitochondrial particles of beef heart (red columns). Solitary columns indicate that the conditions were only tested either for mouse or for beef heart.

Having shown the high sensitivity of the V-ATPase to the two novel antibiotics, we also wanted to estimate their inhibitory effect on F-and P-type ATPases. In various cell cultures growth was inhibited at nanomolar concentrations (see above). Therefore we asked, whether a concentration of 1 μM which is fairly enough to knock out V-ATPase activity and which is clearly higher than the IC_50 _values in the growth experiments, would also affect F-ATPases such as the mitochondrial ATP-synthase or P-ATPases such as the plasma membrane Na^+^/K^+^-ATPase. Thus, we prepared mitochondria rich crude membranes from mouse heart and submitochondrial particles from beef heart as well as highly purified, Na^+^/K^+^-ATPase containing plasma membranes from pig kidney, and tested the inhibitory potential of archazolid and of apicularen. To detect F-ATPase activity we used its specific inhibitors, azide or oligomycin [19], and ouabain or vanadate were used as specific inhibitors of the Na^+^/K^+^-ATPase [20,21]. Whereas the ATPase activity in the mouse and beef heart preparations was reduced to values around 25% by the specific F-ATPase inhibitors azide or oligomycin, the new antibiotics archazolid and apicularen as well as the established specific V-ATPase inhibitors concanamycin and bafilomycin reduced the activity only slightly to approximately 80% (Fig. 4). An increase of the inhibitor concentration to 10 μM for archazolid and apicularen had no substantial effect. As shown in Tab. 2, Na^+^/K^+^-ATPase activity was completely inhibited by 1 mM vanadate and by 1 mM ouabain, whereas it was only slightly affected by apicularen A and by archazolid A. A slight inhibition of comparable size was also observed for the established inhibitor of V-ATPases, concanamycin A, corroborating the well known fact that plecomacrolidic antibiotics start to inhibit P-ATPases at concentrations in the micromolar range [6,7]. Taken together our results with F-and P-ATPases strongly suggest that archazolid and apicularen are specific V-ATPase inhibitors.

**Table 2:** Inhibition of Na^+^/K^+^-ATPase from pig kidney by antibiotics

	specific activity μmol*mg^-1 ^*min^-1^	
	
	experiment 1	experiment 2
control without inhibitors	19.0	15.9
vanadate 0.1 mM	2.0	n. d.
vanadate 1 mM	0	0
ouabain 0.1 mM	5.2	n. d.
ouabain 1 mM	0	0
concanamycin A 1 μM	16.7	12.7
apicularen A 1 μM	17.3	13.2
archazolid A 1 μM	17.1	12.2

### Archazolid and apicularen bind to different parts of the V-ATPase

**Figure 5:**
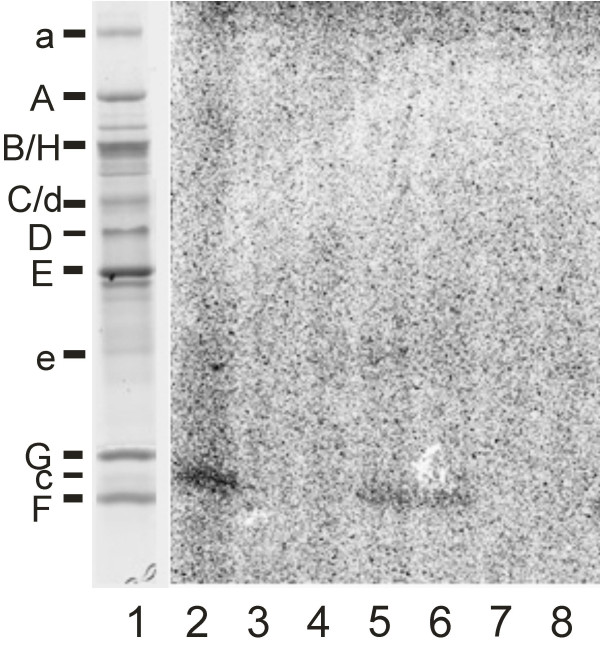
**Protection of the binding site for I-concanolide A by plecomacrolidic antibiotics. **Tricine-SDS-PAGE gels. *Lane 1*, stained with Coomassie Blue; *lane 2–8*, autoradiography of the gel after exposition to a phosphoscreen. Samples of 20 μg V-ATPase were preincubated for 60 min on ice with 100 μM or 10 μM bafilomycin B_1 _(*lanes 3 and 4*), 100 μM or 10 μM apicularen A (*lanes 5 and 6*) and 100 μM or 10 μM archazolid (*lanes 7 and 8*), respectively. I-concanolide A was then added to give a final concentration of 10 μM. The mixture was incubated for another 60 min on ice and then treated with UV light. Control with pre-incubation, but without effectors (*lane 2*).

To investigate the mode of V-ATPase inhibition in more detail, we used the semi synthetic derivative of concanamycin, 9-*O*-[*p*-(trifluoroethyldiazirinyl)benzoyl]-21,23-dideoxy-23-[^125^I]iodo-concanolide A (I-concanolide A, Fig. 1) which already had been used successfully to identify the binding site of plecomacrolides in V-ATPases unambiguously [10]. In our previous study we had shown that the V_o _subunit c is the only subunit of the V-ATPase which is labelled by I-concanolide A and that this labelling could be prevented by pre-incubation with the plecomacrolides concanamycin A, bafilomycin A_1 _and B_1_. In a comparable UV-radiation experiment labelling of subunit c by I-concanolide A was completely abolished by pre-incubation with archazolid A (Fig. 5), thus indicating that plecomacrolides and archazolid A share, at least partially, the same binding site in subunit c.

Unlike archazolid, the benzolactone enamide apicularen A did not appear to interfere with labelling of subunit c by the concanolide A derivative because the signal obtained was almost as strong as in the control (Fig. 4). Therefore we suggest that apicularen binds to a site which is largely different from that for the plecomacrolides. This result is in agreement with our former observation that the benzolactone enamide salicylihalamide does not bind to subunit c and supports our conclusion that the sites and mechanisms of inhibition for benzolactone enamides are different from those for plecomacrolides [10].

## Conclusion

The novel antibiotics archazolid and apicularen are highly efficient and specific novel inhibitors of V-ATPases. Despite the different structures of archazolid and the plecomacrolides they probably have a similar mode of inhibition and their binding sites in the V-ATPase have at least a considerable overlap. In contrast, apicularen which demonstrates the same inhibition efficacy does not interfere with I-concanolide A, thus suggesting a mode of inhibition different from that of the plecomacrolides.

## Methods

### Enzyme preparations

The V-ATPase holoenzyme was purified as published elsewhere [10]. Preparation of highly purified membranes containing Na^+^/K^+^-ATPase from pig kidney followed the protocol of Jørgensen [22] with the three main steps of differential centrifugation, incubation with SDS in the presence of ATP and sucrose density gradient centrifugation in a fixed angle rotor, and led to a specific enzyme activity which was in the same range as that reported by the authors. The resulting sample was stored frozen at -20°C. To prepare crude membrane extracts, the hearts of three mice were washed three times with an ice-cold buffer of pH 8.1 consisting of 0.25 M sucrose, 5 mM Tris-HCl, 5 mM EDTA, and 10 mM Pefabloc SC (Biomol), homogenized in 5 ml of this buffer and centrifuged at 4°C for 25 min at 233,000 × *g*_*max *_in a fixed angle rotor. The resulting pellet was resuspended in 5 ml of a buffer of pH 7.5, consisting of 5 mM Tris-MOPS (3-morpholinopropanesulfonic acid), 10 mM NaCl, 10 mM Pefabloc SC, 9.6 mM 2-mercaptoethanol, 0.53 mM EGTA and 0.1 % Triton X-100. After an aliquot for protein determination had been taken, 10% bovine serum albumin (final concentration) was added to the suspension which was then stored on ice until the activity assays were run. Beef heart mitochondria were isolated by differential centrifugation, following the protocol of Smith by using a blender to homogenize the heart mince [23]. The initial homogenization buffer consisted of 250 mM saccharose, 10 mM KH_2_PO_4_, 10 mM Tris, 2 mM EGTA, 2 mM MgCl_2_, pH 7.4, and further isolation procedures were carried out in the same medium without EGTA [24]. Submitochondrial particles were obtained by ultrasonic treatment of the mitochondria.

### Antibiotics

Labelled I-concanolide A was synthesized as described elsewhere [25]. Archazolid A and B, apicularen A and B, concanamycin A and bafilomycin A_1 _and B_1 _were isolated according to published procedures [15-17,26]. To avoid freeze-thaw cycles which have significant influence on the stability of the substances, aliquots of stock solutions in dimethyl sulfoxide were stored at -70°C and thawed only once immediately before use. The actual concentrations of the stock solutions were determined spectrophotometrically.

### ATPase assays

Standard V-ATPase assays with a final volume of 160 μl and a pH of 8.1 consisted of 3–4 μg of protein, 50 mM Tris-MOPS, 3 mM 2-mercaptoethanol, 1 mM MgCl_2_, 20 mM KCl, 0.003% C_12_E_10_, 20 mM NaCl, and 3 mM Tris-HCl. After 5 min of pre-incubation at 30°C with or without inhibitors, 1 mM Tris-ATP was added and after incubation for 2 min the reaction was stopped by placing the tube in liquid nitrogen. Assays using Na^+^/K^+^-ATPase were performed in 160 μl at pH 7.5 and contained 0.5 μg of protein, 50 mM Tris-MOPS, 5 mM imidazole, 0.2 mM EDTA, 4 mM MgCl_2_, 20 mM KCl and 100 mM NaCl. After 5 min of pre-incubation at 37°C with or without inhibitors, 3 mM Tris-ATP was added and after 1 min of incubation the reaction was stopped by placing the tube in liquid nitrogen. Assays using crude membranes from mouse heart had a volume of 160 μl and a pH of 7.5, and consisted of 10 μg of membrane protein, 50 mM Tris-MOPS, 3 mM 2-mercaptoethanol, 1 mM MgCl_2_, 20 mM KCl, 100 mM NaCl, 0.02% Triton X-100 and 0.3 mg/ml BSA. After 5 min of pre-incubation at 30°C with or without inhibitors, 1 mM Tris-ATP was added and after an incubation time of 10 min the reaction was stopped by placing the tubes in liquid nitrogen. ATPase assays with submitochondrial particles from beef heart were performed in a final volume of 1 ml and a pH of 8.0. The samples contained 9–12 μg protein, 50 mM Tris, 50 mM KCl and 2.5 mM MgCl_2_. After 5 min of preincubation with or without inhibitors, 5 mM ATP was added, and after an additional incubation time of 15 min the reaction was stopped by the addition of 0.4 ml of 20 % TCA.

Inorganic phosphate produced in the assays of V-ATPase, Na^+^/K^+^-ATPase and mouse heart F-ATPase was measured according the protocol of Wieczorek *et al*. [27], while the determination of inorganic phosphate produced in the assays of beef heart F-ATPase followed the method of Fiske and Subarrow [28] using ascorbic acid as reducing agent.

### Labelling

Twenty micrograms of the samples were pre-incubated with the inhibitors for 1 h on ice. The labelled I-concanolide A was then added to give a final concentration of 10 μM. Controls were run without effectors. Mixtures with volumes of 40 μl were incubated for an additional 1 h on ice and then treated for 3 min with UV light (366 nm) on ice. After UV irradiation, 10 μl of 5-fold sample buffer [10] was added, the mixture was heated for 45 s at 95°C, cooled on ice, and subjected to Tricine-SDS-PAGE (16.5% T, 3% C separating gel and 10%T, 3% C spacer gel [29]), followed by Coomassie staining. The gels were sealed in plastic wrap before they were exposed to a phosphoscreen for up to 72 h and analyzed with the aid of a phosphoimager (Molecular Dynamics).

### Cell culture and growth inhibition assay

Cell lines were obtained from the German Collection of Microorganisms and Cell Cultures (DSMZ). Cell lines 3Y1 and M1 (embryonal fibroblasts from 129/Ola × C57BL/6 mice, [30]) were a generous gift from Dr. S. Miyamoto, Fukoka, Japan, and Dr. P. F. Mühlradt, Braunschweig, Germany. All cell lines were cultivated under conditions recommended by the supplier. Growth inhibition was measured in microtiterplates. Aliquots of 120 μl of the suspended cells (50,000/ml) were given to 60 μl of a serial dilution of the inhibitor. After 5 days, growth was determined using the MTT assay [31].

### Cell staining

PtK_2 _(ATCC CCL-56) or KB-3-1 cells were grown on glass coverslips (13 mm diameter) in four-well-plates. Exponentially growing cells were incubated with the inhibitors for 4 hours and stained for lysosomes with 50 nM LysoTracker Red DND-99 and for mitochondria with 75 nM MitoTracker Green FM (both from Molecular Probes) at 37°C for 30 min. The nuclei were stained using Hoechst 33258 (5 μg/ml). The coverslips were mounted upside down in PBS, fixed with nail polish, and observed under the fluorescent microscope.

### Other procedures

Fifth instar larvae of *M. sexta *(Lepidoptera, Sphingidae), weighing 6–8 g, were reared under long day conditions (16 h of light) at 27°C using a synthetic diet modified according to Bell *et al *[32].

## Authors' contributions

MH purified the V-ATPase and the Na^+^/K^+^-ATPase, carried out the enzyme and labelling assays, and drafted the manuscript. FS did the fermentation of the archazolids and carried out the cell culture studies. BK did the fermentation of the apicularens and tested F-ATPase activity in beef heart mitochondria. RJ isolated apicularens. HS isolated archazolids. GI and AZ purified the plecomacrolides and synthesized the I-concanolide A. HW participated in the conception and design of the study and drafted the manuscript. All authors read and approved the final manuscript.
